# Active and Repressive Chromatin-Associated Proteome after MPA Treatment and the Role of Midkine in Epithelial Monolayer Permeability

**DOI:** 10.3390/ijms17040597

**Published:** 2016-04-20

**Authors:** Niamat Khan, Christof Lenz, Lutz Binder, Dasaradha Venkata Krishna Pantakani, Abdul R. Asif

**Affiliations:** 1 Institute for Clinical Chemistry/UMG-Laboratories, University Medical Center, Robert-Koch-Str. 40, 37075 Göttingen, Germany; niamat.khattak@gmail.com (N.K.); christof.lenz@mpibpc.mpg.de (C.L.); lutz.binder@med.uni-goettingen.de (L.B.); krishna.if1@gmail.com (D.V.K.P.); 2 Department of Biotechnology & Genetic Engineering, Kohat University of Science and Technology, Kohat 26000, Khyber Pakhtunkhwa, Pakistan; 3 Bioanalytical Mass Spectrometry, Max Planck Institute for Biophysical Chemistry, Am Fassberg 11, 37077 Göttingen, Germany

**Keywords:** mycophenolic acid, midkine, iMDK, tight junctions

## Abstract

Mycophenolic acid (MPA) is prescribed to maintain allografts in organ-transplanted patients. However, gastrointestinal (GI) complications, particularly diarrhea, are frequently observed as a side effect following MPA therapy. We recently reported that MPA altered the tight junction (TJ)-mediated barrier function in a Caco-2 cell monolayer model system. This study investigates whether MPA induces epigenetic changes which lead to GI complications, especially diarrhea. Methods: We employed a Chromatin Immunoprecipitation-O-Proteomics (ChIP-O-Proteomics) approach to identify proteins associated with active (H3K4me3) as well as repressive (H3K27me3) chromatin histone modifications in MPA-treated cells, and further characterized the role of midkine, a H3K4me3-associated protein, in the context of epithelial monolayer permeability. Results: We identified a total of 333 and 306 proteins associated with active and repressive histone modification marks, respectively. Among them, 241 proteins were common both in active and repressive chromatin, 92 proteins were associated exclusively with the active histone modification mark, while 65 proteins remained specific to repressive chromatin. Our results show that 45 proteins which bind to the active and seven proteins which bind to the repressive chromatin region exhibited significantly altered abundance in MPA-treated cells as compared to DMSO control cells. A number of novel proteins whose function is not known in bowel barrier regulation were among the identified proteins, including midkine. Our functional integrity assays on the Caco-2 cell monolayer showed that the inhibition of midkine expression prior to MPA treatment could completely block the MPA-mediated increase in barrier permeability. Conclusions: The ChIP-O-Proteomics approach delivered a number of novel proteins with potential implications in MPA toxicity. Consequently, it can be proposed that midkine inhibition could be a potent therapeutic approach to prevent the MPA-mediated increase in TJ permeability and leak flux diarrhea in organ transplant patients.

## 1. Introduction

Mycophenolic acid (MPA) is a reversible yet highly selective non-competitive inhibitor of the inosine 5′-monophosphate dehydrogenase-2 (IMPDH-2) enzyme. IMPDH-2 is expressed in proliferating immune cells (B and T cells) and is involved in the biosynthesis of the guanine nucleotide via a *de novo* pathway [1,2]. This inhibitory property led to MPA to be classified as an immunosuppressant which is prescribed to organ transplant patients to prevent graft rejection [3]. Like other commercially available immunosuppressants, MPA is associated with gastrointestinal (GI) complications including diarrhea [4]. Hence, more attention is needed either to focus on dose-related strategies or to explore mechanisms of action that can help to formulate ways to minimize MPA-associated side effects in organ transplant patients.

Drugs may alter the epigenetic status of exposed cells via direct or indirect mechanisms [5]. For example, Hydralazine, which is prescribed to treat hypertension, is known to inhibit DNA methylation in T cells and to induce autoreactivity as a side effect [6]. While acute exposure to isotretinoin, a drug prescribed to treat severe acne as well as various skin cancers, influences the transcriptional activity of transcriptional factor FoxO1. FoxO1 activates the promoter of FoxO1-binding site genes via all-trans-retinoic acid and consequently causes many side effects such as hepatotoxicity, hair loss, bone toxicity, hypertriglyceridemia, *etc.* [5,7]. Recently, it has been reported that MPA downregulates histone deacetylases (Histone deacetylase 2 (HDAC2), Histone Deacetylase 7 (HDAC7) and Sirtuin 1 (SIRT1)) and upregulates histone acetyltransferases (CREB Binding Protein (CREBBO) and P300/CBP-associated factor (PCAF)) in CD4^+^ T cells isolated from systemic lupus erythematosus patients [8]. Therefore, we hypothesized that epigenetic changes may be involved in the etiology of GI complications observed in organ transplant patients after MPA therapy.

The main objective of this study was to correlate the pathophysiology of GI complications, especially MPA-induced diarrhea, in organ transplanted patients, with the altered expression of Chromatin Immunoprecipitation-O-Proteomics (ChIP-O-Proteomics) precipitated protein(s). Our ChIP-O-Proteomics results indicate that MPA treatment increases the expression of midkine as well as its association with H3K4me3-marked chromatin. Functional analysis of a differentiated and polarized Caco-2 cells monolayer revealed that inhibitor of Midkine (iMDK) can significantly inhibit the MPA-mediated increase in monolayer permeability.

## 2. Results and Discussion

**Figure 1:**
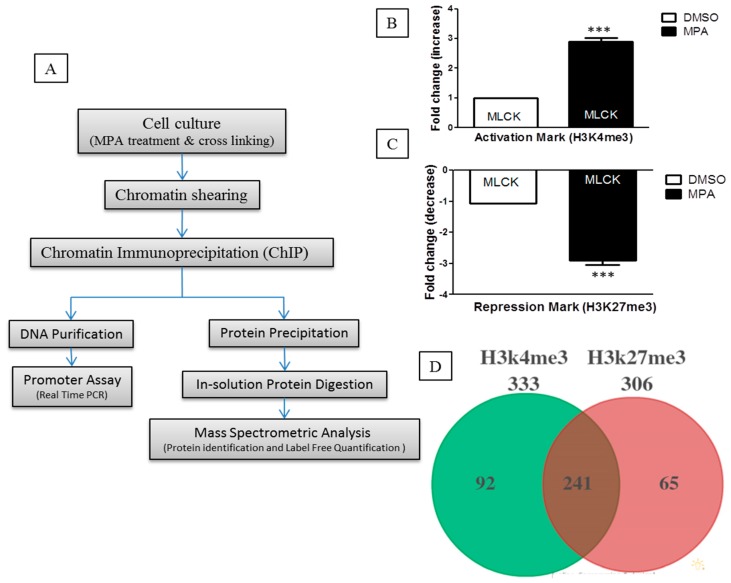

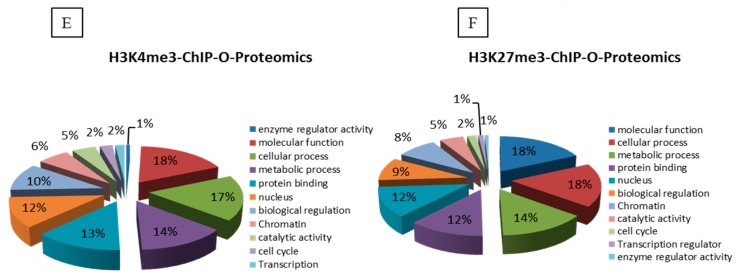
(**A**) Schematic overview of Chromatin Immunoprecipitation-O-Proteomics (ChIP-O-Proteomics) experimental steps. Differentiated and polarized Caco-2 cell monolayers were incubated with MPA (10 µM) or DMSO for 72 h. Sheared chromatin having an active (H3K4me3) or repressive histone modification mark (H3K27me3) was precipitated using antibodies, anti-H3K4me3 or anti-H3K27me3, respectively. IgG was used as a background. Each sample was aliquoted into two portions. One portion was used for DNA-based promoter activity using real-time PCR. The other portion was used to identify and quantify promoter binding proteins using mass spectrometry. Three biological replicates, each one with at least two technical replicates, were performed for each group; (**B**,**C**) ChIP-DNA was subjected to real-time PCR with a specific primer pair of the *MLCK* promoter region. Each bar represents abundance or depletion of the activation mark (H3K4me3) and/or repression mark histone mark (H3K27me3) in the promoter of the *MLCK* gene. Data is presented as a fold change by taking the control as 1.0 and the error bar indicate means ± SEM. *** *p* < 0.001 compared with the control (DMSO); (**D**) Venn diagram show the number of identified proteins associated with an active promoter and repressive promoter following the subtraction of IgG precipitated proteins as background; (**E**,**F**) scaffold analysis of GO for the ChIP-O-Proteomics–identified proteins; (**E**) H3K4me3-associated proteins and (**F**) H3K27me3-associated proteins. Pie charts represent the percentages of the identified proteins belonging to different function groups.

We recently reported that MPA regulates the MLCK and MLC-2 (Myosin Light Chain Kinase and Myosin Light Chain-2) pathway in Caco-2 cell monolayers [9]. Our subsequent results showed that MPA treatment alters the expression of MLCK-MLC2 by modulating the epigenetic status at their respective promoter regions [10]. To identify the proteins that participate or associate with epigenetic changes occurring at the chromatin level during MPA treatment, we undertook a ChIP-O-Proteomics approach (Figure 1A). Towards this, we first performed ChIP, and the immunoprecipitated sample was aliquoted into two parts prior to separation of the DNA and protein complex. One part was analyzed for *MLCK* promoter epigenetic status (for quality control), while the other part was used for ChIP-O-Proteomics analysis. Traditional ChIP-qPCR revealed that the promoter region of *MLCK*, in MPA-treated Caco-2 cells, is enriched for H3K4me3 (Figure 1B,C), further confirming our gene expression data and allowing us to proceed with the ChIP-O-Proteomics approach. Our promoter assay results of *MLCK* are consistent with these findings [11,12].

We used a label-free, quantitative spectral counting–based ChIP-O-Proteomics approach to identify and quantify the proteins associated with either H3K4me3 (active) or H3K27me3 (repressive) histone marks. Proteins associated with chromatin regions that represent either active or repressive promoters of protein coding genes were identified by mass spectrometry using the following criteria (protein threshold = 99%, minimum number of peptides = 2, peptide threshold = 95%, false discovery rate = 1%) for protein selection (Supplementary Table S1). Analysis of variance (ANOVA) was performed with a multiple testing correction (Hochberg-Benjamini correction) using Scaffold software to obtain a quantitative profile of proteins identified at ≥99% confidence using label-free spectral counting. Proteins precipitated by an IgG control were subtracted from the results list. Our ChIP-O-Proteomics approach identified a catalog of proteins at both the active and/or repressive promoter regions. We identified a total of 333 proteins associated with anti-H3K4me3 and 306 proteins associated with anti-H3K27me3 antibodies in MPA-treated cells as well as in DMSO (control)-treated cells. Among these proteins, 241 were commonly found at both active and repressive chromatin sites (Figure 1D). Ninety-two proteins were found to associate specifically with the active histone modification mark, while 65 proteins remained specific to repressive chromatin (Figure 1D). A number of proteins including Caudal Type Homeobox 1 (Cdx-1), Topoisomerase (DNA) II Alpha (TOP2-A), H2A Histone Family, Member X (H2AFX), Regulator of chromosome condensation 1 (RCC1) and Retinoblastoma Binding Protein 4 (RBBP4) have been previously identified as H3K4me3-associated in mouse embryonic stem cells [13]. Similarly, Interleukin 3 (IL3), Heterogeneous Nuclear Ribonucleoprotein M (HNRPM), 40S ribosomal proteins, 60S ribosomal proteins and Lamina-associated polypeptide 2 (LAP2B) were identified as H3K4me3-associated proteins in HeLa cells [14]. In addition, we report here sets of protein candidates (Supplementary Tables S2–S7) that have not been previously reported to be associated with active or repressive chromatin marks. This discrepancy with other studies may be due to the specific cell system and enrichment scheme used here. Unlike genomics, which is rather constant, proteomics is dynamic in nature [15], differing from cell type to cell type and even within the same cells under different environmental conditions.

Next, we analyzed the differential protein complements associated with the active and repressive chromatin regions of MPA-treated Caco-2 cells (Figure 1E,F). Our mass spectrometry data revealed altered enrichment of 45 candidates at the active chromatin region and seven candidates at the repressive chromatin region in MPA-treated Caco-2 cells as compared to DMSO (control)-treated cells (Supplementary Table S1). These differentially enriched proteins are known to be involved in the regulation of transcription, cell cycle, chromatin structure, chromosome segregation and condensation, while their altered expression results in the etiology of certain diseases (Figure 1E,F).

**Figure 2:**
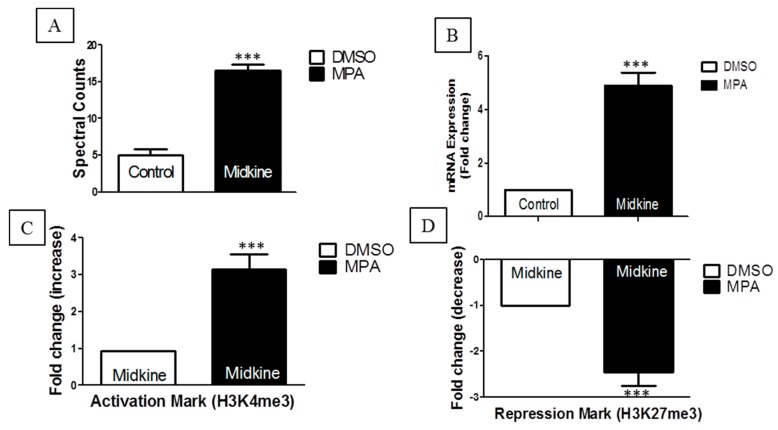

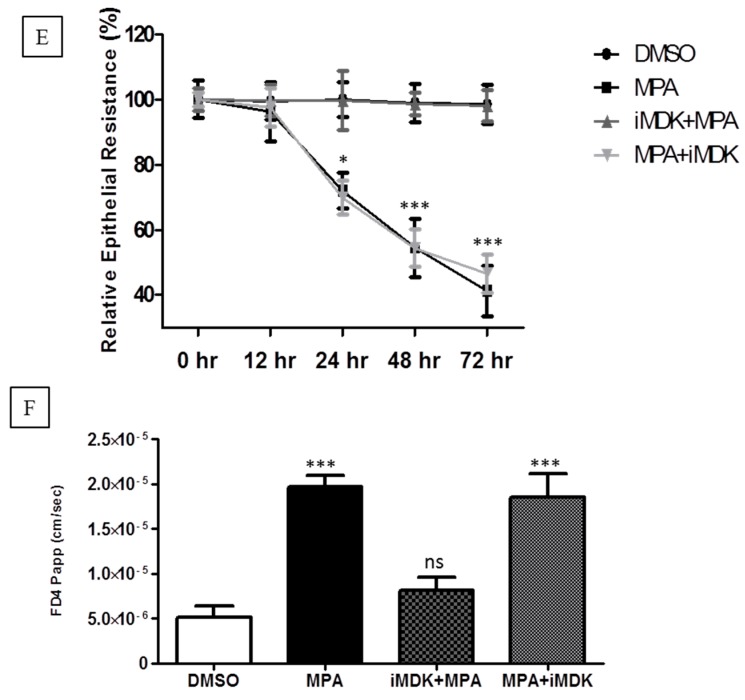
(**A**–**D**) Influence of MPA (10 µM) on the expression and activation of the *midkine* gene. (**A**) Quantification of the midkine protein by spectral count after MPA treatment as compared to DMSO (control) cells; (**B**) expression (mRNA) of the *midkine* gene after MPA treatment. Expression of the mRNA of *midkine* was investigated using quantitative real-time PCR; (**C**,**D**) Influence of MPA treatment on the enrichment or depletion of histone active modification mark (H3K4me3) and repressive histone modification mark (H3K27me3) in the promoter of the *midkine* gene; (**E**,**F**) Caco-2 cell monolayer integrity. Caco-2 cells were seeded on the transwell insert membrane. Differentiated and polarized Caco-2 cells were treated as described in material and method; (**E**) TEER was measured at the indicated times in the graph; (**F**) following 72 h incubation, FD4 dye was added to the apical chamber and dye concentration was measured in the sample collected from the basal chamber. TEER and FITC-dextran assay results obtained either in presence or absence of midkine inhibitor (iMDK, 25 nM). Error bars indicate means ± SEM. * *p* < 0.05, *** *p* < 0.001. Differences between two groups were analyzed by the two-tailed Student’s *t*-test and for more than two groups analysis of variance (ANOVA) was applied with Bonferroni post-test (*n* = 3). ns: non-significant.

Interestingly, our nanoLC-MS/MS results showed significant increases in spectral counts for the midkine protein in MPA-treated cells (Figure 2A). This result was further confirmed at the mRNA (Figure 2B) and epigenetic levels (Figure 2C,D). Midkine is a multifunctional cytokine or growth factor that provokes various biological processes including growth, migration, survival, repair and gene expression [16]. Apart from its normal function, over-expression of midkine is considered to play an important role in the etiology of various diseases such cancer and inflammatory diseases [16]. Elevated concentrations of circulating midkine have been observed in the serum of Crohn’s disease (CD) [17] and ulcerative colitis (UC) [18] patients. Increased TJ permeability via different cytokines is also associated with CD and UC diseases [19]. However, the role of midkine in the regulation of the TJ assembly has not been reported in the context of GI tract complications. To understand whether MPA increases midkine-mediated monolayer permeability, we performed inhibitory analyses of the increased expression of midkine through an iMDK in a MPA-treated Caco-2 cells monolayer. In order to check any possible cytotoxic effects of iMDK on the Caco-2 cell monolayer, the cells were incubated with either MPA alone or in combination with varying concentrations of iMDK and tested for cell viability. These results showed that MPA treatment alone, as well as co-treatment with iMDK (5–25 nM), had no significant cytotoxic effect as compared to the control cells (Supplementary Figure S1A,B) which was further confirmed by measuring the cytotoxicity biomarker lactate dehydrogenase (LDH). However, higher concentrations of iMDK (≥50 nM) caused significant cytotoxicity (Supplementary Figure S1A,B). We further evaluated the inhibitory concentration of iMDK to prevent midkine expression in MPA-treated Caco-2 cells. Our results show that MPA-mediated increased expression of midkine was prevented in Caco-2 cell monolayers by pretreatment with a non-cytotoxic concentration of iMDK (25 nM) (Supplementary Figure S1C). Therefore, we assessed the Caco-2 cell monolayer integrity using TEER and FD4-flux assays in three different treatment groups: (1) MPA (10 µM); (2) iMDK (25 nM) + MPA (10 µM) (iMDK was added 1 h prior to co-incubation with MPA for the next 72 h); (3) MPA (10 µM) + iMDK (25 nM) (MPA treatment for 60 h, followed by co-incubation with iMDK for 12 h). The DMSO-treated monolayer served as a control. TEER and FD4-flux assays are well-established parameters which are traditionally used to assess epithelial monolayer integrity [20]. Epithelial monolayer integrity is lost either to cell death or compromised TJ assembly. TJ is a complex but dynamic structure; different types of stimuli increase TJ permeability via different types of cellular signaling pathways in various disease models [19]. In agreement with our previous results, TEER values of MPA-treated confluent Caco-2 cell monolayers significantly dropped in a time-dependent manner as compared to the control monolayer and, concomitantly, the FD4 dye flux concentration increased across the Caco-2 cell monolayer (Figure 2E,F). However, pretreatment with the iMDK significantly blocked the MPA-mediated decrease of TEER values, and increased the FD4 dye flux across the Caco-2 monolayer (Figure 2E,F). On the other hand, when iMDK was added at the later stage of MPA treatment, no significant differences were observed compared to MPA treatment alone (Figure 2E,F). Although midkine is well known as a cytokine, few studies have reported the localization of midkine to the nucleus as well as to the nucleolus, as well as its involvement in the transcription of 54S rRNA [21,22], supporting our identification of midkine in the chromatin fraction.

The ChIP-O-Proteomics approach is a relatively new but effective technique to identify novel chromatin-associated targets [13,14]. Using our ChIP-O-Proteomics protocol, we could identify a number of new proteins associated with the active and repressive chromatin marks. Furthermore, a semiquantitative spectral counting approach yielded differential enrichment of 45 candidates at the active chromatin region and seven candidates at the repressive chromatin region in MPA-treated cells as compared to the controls. MPA at the used concentration showed no significant cytotoxic effects alone or in combination with the iMDK. The inhibition of midkine significantly blocked the MPA-mediated epithelial monolayer permeability. These results indicate that the combination of immunosuppressive drugs with additional substances such as the iMDK may be helpful in avoiding the unwanted effects of an immunosuppressive regime. However, further detailed *in vivo* and *in vitro* studies are required to make the immunosuppressive regime safer for transplanted patients.

## 3. Material and Methods

### 3.1. ChIP-O-Proteomics

Caco-2 cells were purchased from DSMZ (German collection of microorganisms and cell culture, Braunschweig, Germany) and grown for 13 days post-confluence to establish a monolayer of differentiated and polarized cells resembling enterocytes. Monolayers were incubated with MPA (10 µM) or DMSO (as a control) for 72 h. ChIP assay was performed as following; Caco-2 cells were fixed with 37% formaldehyde at a final concentration of 1% for 30 min at room temperature (RT) followed by quenching with glycine (0.125 M) for an additional 5 min at RT. Cells were collected in ice-cold PBS and equal numbers of cells from each treatment group were lysed with lysis buffer (Red ChIP Kit™, Diagenode Inc., Sparta, NJ, USA). Chromatin was sheared using a Branson Sonifier 250 in shearing buffer according to the manufacturer’s instructions (Red ChIP Kit™, Diagenode Inc., Sparta, NJ, USA). After pre-clearing, chromatin from each treatment was divided into three aliquots. An aliquot from each sample was either incubated with active histone mark antibody (anti-H3K4me3; Abcam, Cambridge, UK) or repressive histone mark antibody (anti-H3K27me3; Millipore, Temecula, CA, US), or with IgG (One Day Kit™, Diagenode Inc., Denville, NJ, USA) for overnight at 4 °C. Antibody-chromatin complexes were further incubated with pre-activated Protein-A magnetic beads, washed and sample was divided into two portions. One portion was treated with proteinase-K to use for a promoter study at DNA level and the other portion was used for the identification of chromatin binding proteins using mass spectrometry.

### 3.2. Promoter Assay

DNA-protein cross-linking was reversed by degrading DNA binding proteins with proteinase K. DNA was purified with DNA slurry (One Day Kit™, Diagenode Inc., Denville, NJ, USA) and quantified by NanoDrop (NanoDrop 2000C, Thermo Scientific, peqlab Biotechnologie GmbH, Erlangen, Germany). ChIP-precipitated genomic DNA was used as a template in a SYBR green (Roche, Manheim, Germany) based real time PCR reaction. Comparative threshold cycle (*C*_t_) method (2^–ΔΔ*C*t^) [23] was used to analyze the real time PCR data and described as fold change. Data was normalized to *GAPDH*, a reference gene.

### 3.3. Proteins Purification from Chromatin-Antibody Complexes

Glycine elution method was used to isolate proteins from chromatin-antibody complexes. Briefly, glycine buffer (pH 2.5) was added to each sample for 5 min with agitation at RT. Supernatant was neutralized by adding Tris (pH 8.0). To eliminate interfering substances such as detergents, salts, phenolic acid and nucleic acid, proteins were precipitated overnight by acetone precipitation and the pellets was dried in a SpeedVac concentrator (UNIVAPO 150 H, Uniequip, Martinsried, Germany). Proteins were then reduced with 25 mM Dithiothreitol (DTT), alkylated by adding 100 mM Iodoacetamide (IAA) and subject to in solution trypsin digestion. To stop the trypsin activity each sample was acidified by incubating with 5% Trifluoroacetic acid (TFA) and the supernatant was collected, dried using a SpeedVac, reconstituted in 2% acetonitrile/0.1% formic acid (*v*:*v*) and prepared for nanoLC-MS/MS analysis.

### 3.4. NanoLC-MS/MS Analysis

For mass spectrometric analysis, samples were enriched on a self-packed reversed phase-C18 precolumn (0.15 mm ID × 20 mm, Reprosil-Pur120 C18-AQ 5 µm, Dr. Maisch HPLC GmbH, Ammerbuch-Entringen, Germany) and separated on an analytical reversed phase-C18 column (0.075 mm ID × 200 mm, Reprosil-Pur 120 C18-AQ, 3 µm, Dr. Maisch HPLC GmbH (Ammerbuch-Entringen, Germany) using a 30 min linear gradient of 5%–35% acetonitrile/0.1% formic acid (*v*:*v*) at 300 nL·min^–1^. The eluent was analyzed on a Q Exactive hybrid quadrupole/orbitrap mass spectrometer (ThermoFisher Scientific, Dreieich, Germany) equipped with a FlexIon nanoSpray source and operated under Excalibur 2.4 software using a data-dependent acquisition method. Each experimental cycle was of the following form: one full MS scan across the 350–1600 *m*/*z* range was acquired at a resolution setting of 70,000 Full Width Half Maximum (FWHM), and automatic gain control (AGC) target of 1 × 10^6^ and a maximum fill time of 60 ms. Up to the 12 most abundant peptide precursors of charge states 2 to 5 above a 2 × 10^4^ intensity threshold were then sequentially isolated at 2.0 FWHM isolation width, fragmented with nitrogen at a normalized collision energy setting of 25%, and the resulting product ion spectra recorded at a resolution setting of 17,500 FWHM, and AGC target of 2 × 10^5^ and a maximum fill time of 60 ms. Selected precursor *m*/*z* values were then excluded for the following 15 s. Two technical replicates per sample were acquired.

### 3.5. Data Processing

Peak lists were extracted from the raw data using Raw2MSMS software v1.17 (Max Planck Institute for Biochemistry, Martinsried, Germany). Protein identification was achieved using MASCOT 2.4 software (Matrixscience, London, UK). Proteins were identified against the UniProtKB *Homo sapiens* reference proteome v2015.02 (20,268 protein entries along with a set of 51 contaminants commonly identified in our laboratory). The search was performed with trypsin as enzyme and iodoacetamide as cysteine blocking agent. Up to two missed tryptic cleavages and methionine oxidation as a variable modification were allowed for. Search tolerances were set to 10 ppm for the precursor mass and 0.05 Da for fragment masses, and ESI-QUAD-TOF specified as the instrument type.

Scaffold software version 4.4.1.1 (Proteome Software Inc., Portland, OR, USA) was used to validate MS/MS based peptide and protein identifications. Peptide identifications were accepted if they could be established at greater than 95.0% probability by the Percolator algorithm. Protein probabilities were assigned by the Protein Prophet algorithm [24]. Protein identifications were accepted if they could be established at greater than 99% by the Percolator algorithm and contained at least two identified peptides. Protein hits that contained similar peptides and could not be differentiated based on MS/MS analysis alone were grouped to satisfy the principles of parsimony. Proteins sharing significant peptide evidence were grouped into clusters. Proteins were annotated with Gene Ontology (GO) terms using the NCBI library downloaded on 23 February 2015 [25]. Relative quantification of proteins in the samples was achieved by analysis of variance (ANOVA) of normalized Spectral Counts using a Benjamini-Hochberg-corrected *p*-value of 0.1 to judge significance. To allow for the calculation of low abundance protein ratios, a value of three spectral counts was introduced as a plausible minimum where necessary to avoid division by zero issues.

### 3.6. mRNA Expression Assay

Caco-2 cells were grown and treated as previously described [9,10]. Briefly, differentiated and polarized Caco-2 cells monolayers were treated with MPA or iMDK (inhibitor of Midkine “3-(2-(4-fluorobenzyl)imidazo[2,1-b]thiazol-6-yl)-2H-chromen-2-one” Axon Medchem BV, The Netherlands) + MPA or MPA + iMDK or DMSO (control) for 72 h. Cells were harvested and the total RNA was isolated using the Trizol method. Total RNA was quantified using (NanoDrop 2000C, Thermo Scientific, peqlab, Biotechnologie GmbH, Erlangen, Germany), reverse transcribed and the expression of the *midkine* gene was quantified by real time PCR using SYBRGreen as the detecting dye as previously described [10,26,27]. *GAPDH* was used as reference gene to normalize the data.

### 3.7. Cell Cytotoxicity Assays

Differentiated and polarized monolayers of Caco-2 cells were treated with MPA or iMDK (0–100 nM) + MPA or DMSO for 72 h (Supplementary Figure S1). Following 72 h of treatment, supernatants were collected to measure the lactate dehydrogenase (LDH) marker using cytotoxicity detection kit (LDH FS, DiaSys, Diagnostic Systems GmbH, Holzheim, Germany) according to the manufacturer’s guidelines. Cells were collected and the trypan blue exclusion test performed to assess cell viability as previously described [13,14,15].

### 3.8. Caco-2 Monolayer Integrity

Transepithelial electrical resistance (TEER) and FITC-dextran (FD4) dye (1 mg/mL) assays were performed as described previously [12] to assess the intactness of the paracellular pathway in the monolayer between adjacent cells. Briefly, differentiated and polarized monolayers of Caco-2 cells were established on transwell insert membranes. Each monolayer was treated with MPA or iMDK + MPA or MPA + iMDK or DMSO (control) for 72 h. TEER values were recorded at several indicated time points. Following 72 h treatment and TEER reading, each monolayer was washed and FD4 dye was added to the apical chamber for 2 h. Samples were collected from basolateral chamber and dye concentrations were measured using a Lambda fluoro 320 fluorescence plate reader (MWG Biotech, Ebersberg, Germany).

## Supplementary Materials

Supplementary materials can be found at http://www.mdpi.com/1422-0067/17/4/597/s1.
