# Supplementary material for: Active and Repressive Chromatin-Associated Proteome after MPA Treatment and the Role of Midkine in Epithelial Monolayer Permeability
Source: Int J Mol Sci. 2016 Apr 20;17(4):597. doi: 10.3390/ijms17040597 (PMC4849051; doi:10.3390/ijms17040597)
Supplement: Supplementary file 1 [file ijms-17-00597-s001.zip › ijms-121525-Supplementary Materials/ijms-121525-supplementary Figure S1.pdf]

Supplementary Materials: Active and Repressive Chromatin Associated Proteome after MPA Treatment and the Role of Midkine in Epithelial Monolayer Permeability

Niamat Khan, Christof Lenz, Lutz Binder, Dasaradha Venkata Krishna Pantakani and Abdul R. Asif

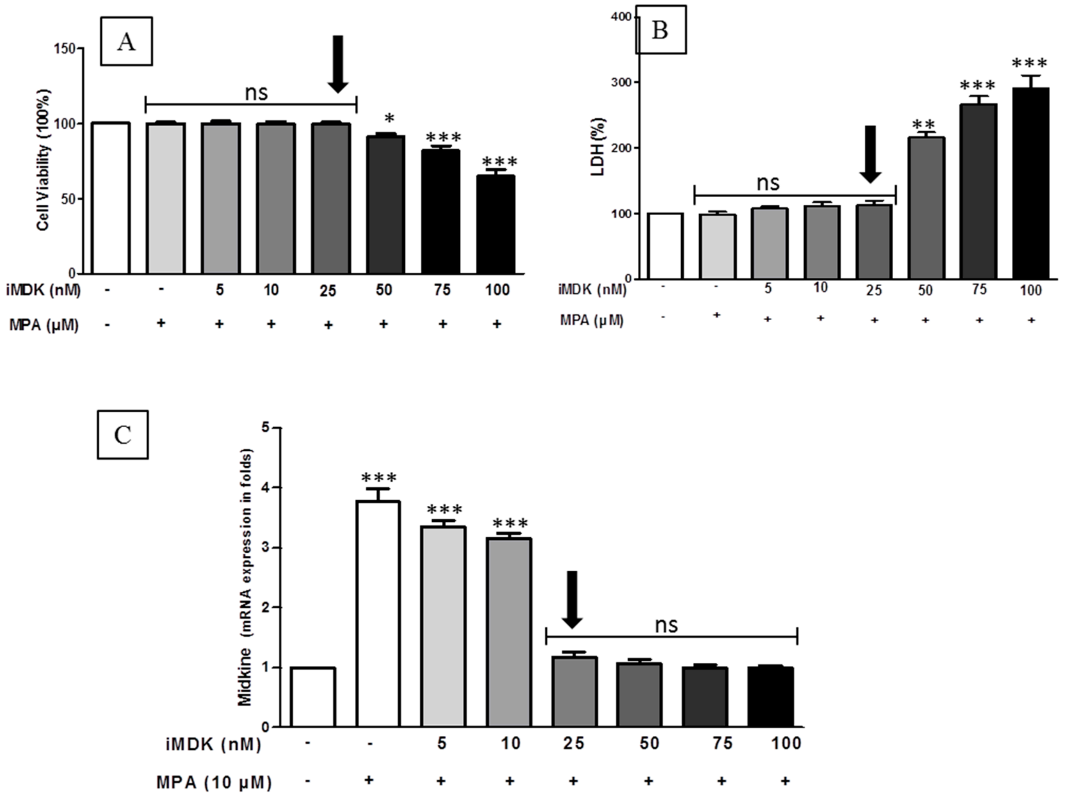

Figure S1. Influence of midkine inhibitor (iMDK) on cell viability and midkine expression of MPA-treated Caco-2. (**A**) Viability of Caco-2 cells (trypan blue dye exclusion assay) after 72 h treatment with DMSO (control) and MPA (10 µM) alone or in combination with different concentration of iDMK inhibitor (0–100 nM). Cell viability was markedly decreased when Caco-2 cells were co-incubated with iMDK (≥50 nM) plus MPA (10 µM); (**B**) cytotoxic effects of midkine inhibitor were assessed by performing LDH leakage assay. Significant leakage of cytotoxic marker (LDH) was found after iMDK (≥50 nM) plus MPA (10 µM) treatment; (**C**) expression of midkine after 72 h treatment with DMSO (control), MPA (10 µM) alone or in combination with midkine inhibitor (iMDK). iMDK significantly inhibits the expression of midkine gene at the concentration of ≥25 nM. Each bar represents separated experiment. Data are presented as means ± SEM. * $p < 0.05$, ** $p < 0.01$, *** $p < 0.001$ compared with the control (DMSO) at that time by analysis of variance (ANOVA) using a Bonferroni post-test. ($n = 3$). ns: non-significant.
