# Supplementary material for: Active and Repressive Chromatin-Associated Proteome after MPA Treatment and the Role of Midkine in Epithelial Monolayer Permeability
Source: Int J Mol Sci. 2016 Apr 20;17(4):597. doi: 10.3390/ijms17040597 (PMC4849051; doi:10.3390/ijms17040597)
Supplement: Supplementary file 1 [file ijms-17-00597-s001.zip › ijms-121525-Supplementary Materials/ijms-121525-supplementary Table S1 .pdf]

Supplementary Materials: Active and Repressive Chromatin Associated Proteome after MPA Treatment and the Role of Midkine in Epithelial Monolayer Permeability

Niamat Khan, Christof Lenz, Lutz Binder, Dasaradha Venkata Krishna Pantakani and Abdul R. Asif

Table S1. List of significantly altered proteins associated with active (H3K4me3) and or repressive (H3K27me3) histone modification marks in MPA treated Caco-2 cells.

ChIP	Accession No.	Protein Name	Fold Change	*p*-Value	Functions (Uniprot)
**Histone Modification Active Mark (H3K4me3)**	Q9UN86	Ras GTPase-activating protein-binding protein 2 OS	2.5 ↓	0.0067	mRNA transportation
	P63104	14-3-3 protein zeta/delta	1.1 ↑	0.0031	Adaptor protein
	P61978	Heterogeneous nuclear ribonucleoprotein K	1.4 ↑	0.0032	Transcription activation/repression, hnRNAs metabolism
	P07355	Annexin A2	1.2 ↑	0.0011	Heat response
	P68104	Elongation factor 1-alpha 1	1.1 ↑	0.0011	Transcription activity, protein synthesis
	Q8IUE6	Histone H2A type 2-B	1.2 ↑	0.0087	Chromatin structure
	P62258	14-3-3 protein epsilon	1 ↑	0.005	Adaptor protein
	P61254	60S ribosomal protein L26	1 ↑	0.0078	
	Q01844	RNA-binding protein EWS	1.1 ↑	0.003	Repressor
	P68431	Histone H3.1	1.4 ↑	0.0011	Chromatin structure
	P16402	Histone H1.3	1.1 ↑	0.00019	Chromatin structure, regulator of individual gene transcription
	P62249	40S ribosomal protein S16	1.25 ↓	0.0042	
	Q15691	Microtubule-associated protein RP/EB family member 1	1.1 ↓	0.0046	Microtubule base cytoskeleton
	Q13242	Serine/arginine-rich splicing factor 9	1.1 ↑	0.00038	Splicing activity
	P37108	Signal recognition particle 14 kDa protein	1.0 ↑	0.0039	
	Q96AE4	Far upstream element-binding protein 1	1.4 ↑	0.0063	Transcriptional activity
	P24534	Elongation factor 1-beta	1.3 ↑	0.0015	Exchanging EF-1alpha bound GDP to GTP
	Q12906	Interleukin enhancer-binding factor 3	1.5 ↑	0.00042	Gene regulation, protein synthesis
	P10599	Thioredoxin	1.1 ↑	0.00048	Transcription activity, redox reaction
	P62081	40S ribosomal protein S7	1.3 ↑	0.0071	rRNA maturation
	P62851	40S ribosomal protein S25	0.9 ↓	0.0006	
	Q12874	Splicing factor 3A subunit 3	1.3 ↑	0.0058	Subunit of A & E complex
	P62857	40S ribosomal protein S28	1.1 ↓	0.0043	
	P09234	U1 small nuclear ribonucleoprotein C	1.1 ↑	0.0054	Splicing activity
	Q14157	Ubiquitin-associated protein 2-like	1.3 ↑	0.00014	Ubiquitin-proteasome pathway, growth and migration of prostate cancer cells
	P25398	40S ribosomal protein S12	1.25 ↓	0.0082	
	Q99459	Cell division cycle 5-like protein	1.6 ↑	0.0032	Cell cycle regulator, transcription activity
	O95793	Double-stranded RNA-binding protein Staufen homolog 1	1.5 ↑	0.0028	Cross linking cytoskeleton, RNA component, translation
	P62424	60S ribosomal protein L7a	1.3 ↑	0.0074	
	Q92522	Histone H1x	1.7 ↑	0.008	Chromatin condensation
	Q71UI9	Histone H2A.V	1.2 ↑	0.0017	Chromosome segregation, cell division
	Q9NYL4	Peptidyl-prolyl *cis*-trans isomerase FKBP11	1.11 ↓	0.0065	Protein folding
	P12277	Creatine kinase B-type	1.4 ↑	0.0096	Energy transduction
	P14866	Heterogeneous nuclear ribonucleoprotein L	1.8 ↑	0.0022	Splicing activity, regulator of exon inclusion
	P02545	Prelamin-A/C	1.7 ↑	0.0022	Nuclear lamina, chromatin organization, telomere dynamics
	P20700	Lamin-B1	1.8 ↑	0.002	Nuclear lamina, chromatin organization, telomere dynamics
	P23528	Cofilin-1	1.4 ↑	0.0016	Cell morphology, cytoskeletal organization
	Q13813	Spectrin alpha chain, non-erythrocytic 1	5.5 ↑	0.0038	Cytoskeleton
	P21796	Voltage-dependent anion-selective channel protein 1	1.9 ↑	0.0046	Cell volume, apoptosis

Table S1. *Cont.*

ChIP	Accession No.	Protein Name	Fold Change	*p*-Value	Functions (Uniprot)
	Q15365	Poly(rC)-binding protein 1	1.8 ↑	0.0029	Nucleic acid binding protein
	P53999	Activated RNA polymerase II transcriptional coactivator p15	2.1 ↑	0.006	Stabilizing the multiproteins transcription complex
	P09327	Villin-1	2.5 ↑	0.005	Cell morphology, division, migration and apoptosis
	**P21741**	**Midkine**	**3.8 ↑**	**0.00010**	**Growth factor, activator of PI3K, MAPK pathways**
	Q15366	Poly(rC)-binding protein 2	2.2 ↑	0.0093	Nucleic acid binding protein, adaptor & regulator protein
	P61586	Transforming protein RhoA	3 ↑	0.0071	Signal transduction pathway, activator, cell migration
**Histone Modification Repressive Mark (H3K27me3)**	Q06830	Peroxiredoxin-1	1.67 ↓	0.00094	Redox regulation
	P13639	Elongation factor 2	1.67 ↓	0.00010	ribosomal translocation
	P12956	X-ray repair cross-complementing protein 6	5 ↓	0.00098	Helicase activity, chromosome translocation, negative transcription regulators
	Q9ULV4	Coronin-1C	3.33 ↓	0.00044	Cytokinesis, motility, and signal transduction
	P14174	Macrophage migration inhibitory factor	1.25 ↓	0.00058	Pro inflammatory cytokines
	P10599	Thioredoxin	1.1 ↑	0.00010	Redox reactions, DNA binding activity
	Q9NX24	H/ACA ribonucleoprotein complex subunit 2	4.3 ↑	0.00010	Ribosome biogenesis, telomere maintenance

Arrows indicate "↑" upregulation and "↓" downregulation of the respective proteins after MPA treatment as compared with DMSO (control in fold change). Protein highlighted in the **bold** (Midkine) was further characterized by exploring its role in the increased permeability of Tight Junctions in Caco-2 cells monolayer after MPA treatment.
